# Novel CFTR Activator Cact-3 Ameliorates Ocular Surface Dysfunctions in Scopolamine-Induced Dry Eye Mice

**DOI:** 10.3390/ijms23095206

**Published:** 2022-05-06

**Authors:** Dongkyu Jeon, Ikhyun Jun, Ho K. Lee, Jinhong Park, Bo-Rahm Kim, Kunhi Ryu, Hongchul Yoon, Tae-im Kim, Wan Namkung

**Affiliations:** 1 Yonsei Institute of Pharmaceutical Sciences, College of Pharmacy, Yonsei University, 85 Songdogwahak-ro, Yeonsu-gu, Incheon 21983, Korea; armisael1990@gmail.com (D.J.); jason.hoking@gmail.com (H.K.L.); jjinung@yonsei.ac.kr (J.P.); rkh1497@gmail.com (K.R.); 2 Department of Ophthalmology, Yonsei University College of Medicine, 50 Yonsei-ro, Seodaemoon-Gu, Seoul 03722, Korea; hadesdual@yuhs.ac (I.J.); cjlovem@yuhs.ac (B.-R.K.); 3 Research Laboratories, ILDONG Pharmaceutical Co., Ltd., 20, Samsung 1-ro 1-gil, Hwaseong 18449, Korea; hyoon@ildong.com

**Keywords:** CFTR, activator, Cact-3, dry eye disease, scopolamine

## Abstract

Cystic fibrosis transmembrane conductance regulator (CFTR) is highly expressed on the ocular epithelium and plays a pivotal role in the fluid secretion driven by chloride transport. Dry eye disease is one of the most common diseases with limited therapeutic options. In this study, a high-throughput screening was performed to identify novel CFTR activators capable of inducing chloride secretion on the ocular surface. The screening of 50,000 small molecules revealed three novel CFTR activators. Among them, the most potent CFTR activator, Cact-3 (7-(3,4-dimethoxyphenyl)-N-(4-ethoxyphenyl)pyrazolo [1,5-α]pyrimidine-2-carboxamide), produced large and sustained Cl^−^ currents in WT-CFTR-expressing FRT cells with no alterations of ANO1 and hERG channel activity. The application of Cact-3 strongly activated CFTR in the ocular epithelia of mice and it also significantly increased CFTR-mediated Cl^−^ transport in a primary cultured human conjunctival epithelium. Cact-3 strongly stimulated tear secretion in normal mice. In addition, Cact-3 significantly reduced ocular surface damage and the expression of proinflammatory factors, including interleukin (IL)-1β, IL-6, tumor necrosis factor (TNF)-α, and interferon (IFN)-γ in an experimental mouse model of dry eye disease. These results suggest that Cact-3, a novel CFTR activator, may be a potential development candidate for the treatment of dry eye disease.

## 1. Introduction

Dry eye disease (DED) is one of the most prevalent diseases worldwide and is characterized by symptoms of dryness, visual disturbance, irritation, pain, and other inflammation-related symptoms [1,2]. DED caused by a variety of factors, including insufficient tear production, excessive tear evaporation, and inflammation of the ocular surface [3,4]. Currently, artificial tears (which moisturize the eyes for a short period of time) and cyclosporine and lifitegrast (which inhibit T cell activation and cytokine production) are commonly used for DED treatment [5,6,7]. However, these treatments do not satisfy the unmet medical need for effective treatments for the continuously increasing number of patients with DED [8].

In ocular surface epithelial cells, several ion channels such as cystic fibrosis transmembrane conductance regulator (CFTR), anoctamin 1(ANO1), and epithelial Na^+^ channel (ENaC) are expressed on the apical membrane and these ion channels play an important role in the regulation of fluid secretion and absorption in the aqueous tear film layer [9,10,11]. CFTR is functionally expressed in bulbar and palpebral conjunctiva and provides Cl^-^ secretion and the transport of water into the tear film [10,11,12]. Interestingly, CF patients with CFTR defects have ocular surface abnormalities with a low tear film stability; a recent study revealed robust CFTR activity in the ocular surface epithelial lining of normal human subjects, but not in CF patients [13,14,15,16]. In addition, the application of CFTR modulators that induce the activation or potentiation of CFTR on the ocular surface significantly increases tear volume and ameliorated DED in mice models [17,18,19]. These results suggest that the fluid secretion from the ocular epithelia via the activation of CFTR can restore the impaired aqueous tear film layer and reduce the hyperosmolarity of tear fluid, a major cause of ocular surface inflammation in DED. Thus, the application of CFTR activators could be a novel strategy for the treatment of DED.

In this study, we performed a cell-based high-throughput screening to identify potent and selective CFTR activators as potential agents for DED and discovered Cact-3, a novel CFTR activator. The effects of Cact-3 on the channel activity of CFTR, ANO1, and the human ether-a-go-go-related gene (hERG) were observed and the efficacy of Cact-3 was demonstrated in a mouse model of DED.

## 2. Results

### 2.1. Identification of Novel and Potent CFTR Activators

**Figure 1:**
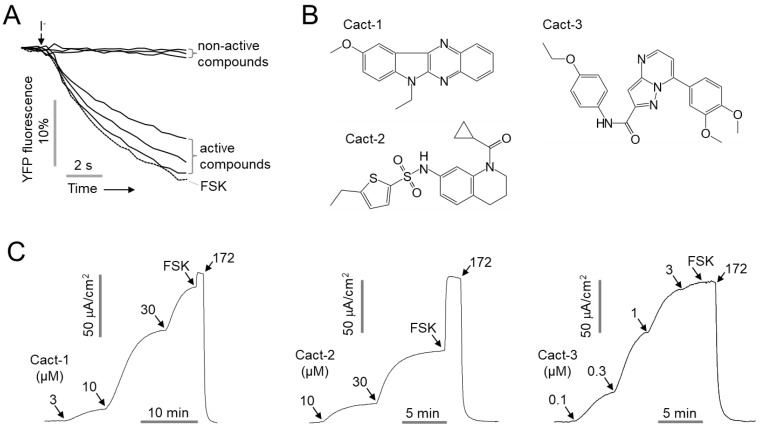
Identification of novel small molecule activators of CFTR. (**A**) Representative traces of YFP fluorescence measured in FRT cells expressing human CFTR. The cells were pretreated with 25 μM of test compounds for 10 min. CFTR was activated by 20 μM forskolin (FSK). (**B**) Chemical structure of novel CFTR activators. (**C**) Apical membrane currents were measured in FRT-CFTR cells in the presence of a transepithelial chloride gradient. CFTR was activated with the indicated concentrations of Cact-1, Cact-2, and Cact-3. CFTR was fully activated by 20 μM forskolin and inhibited by 10 μM CFTR_inh_-172 (172).

For the identification of potent and selective CFTR activators, a cell-based screen was performed using CHO-K1 cells stably transfected with halide-sensing yellow fluorescent protein, YFP-F46L/H148Q/I152L, and human WT-CFTR. The screening of 50,000 small molecules revealed 11 compounds that exhibited > 70% activity compared with the CFTR activity by forskolin (10 μM) at 25 μM. The secondary testing of 11 compounds by an apical membrane current measurement in WT-CFTR-expressing FRT cells showed that only 3 of the 11 compounds induced a CFTR Cl^-^ current (Figure 1). Among the three activators, Cact-3 induced a potent CFTR activation comparable with that by forskolin in the apical membrane current measurement; thus, we further investigated Cact-3.

### 2.2. Cact-3 Potently Activates CFTR Chloride Channel

**Figure 2:**
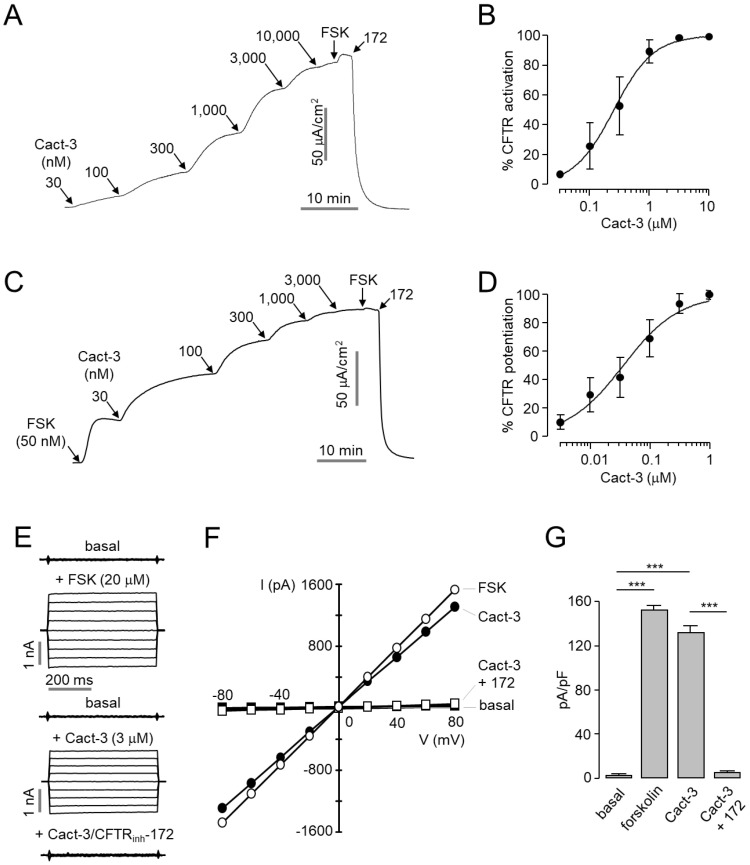
Potent activation of CFTR Cl^-^ channels by Cact-3. (**A**) Apical membrane currents were measured in FRT-CFTR cells. CFTR was activated with the indicated concentrations of Cact-3. (**B**) Summary of CFTR activation (mean ± S.E., *n* = 3). (**C**) Potentiation of CFTR channel activity by the indicated concentrations of Cact-3. CFTR was weakly activated by 50 nM forskolin, fully activated by 20 μM forskolin, and inhibited by 10 μM CFTR_inh_-172. (**D**) Summary of CFTR potentiation (mean ± S.E., *n* = 3). (**E**) Whole-cell currents were recorded at a holding potential of 0 mV and pulsed to voltages between ±80 mV (in steps of 20 mV) in CHO-K1 cells expressing WT-CFTR. CFTR was activated by indicated concentrations of forskolin and Cact-3 and inhibited by 10 μM CFTR_inh_-172. (**F**) Current/voltage(I/V) plot of mean currents at the middle of each voltage pulse. (**G**) Summary of current density at +80 mV (mean ± S.E., *n* = 3). *** *p* < 0.001.

The apical membrane current measurement in WT-CFTR-expressing FRT cells revealed that Cact-3 activated CFTR in a dose-dependent manner with an EC_50_ of 255 nM (Figure 2A,B). Interestingly, Cact-3 not only activated CFTR, but also significantly potentiated the CFTR Cl^-^ current with an EC_50_ of 36.2 nM (Figure 2C,D). As shown in Figure 2E–G, whole-cell patch-clamp measurements showed that CFTR activation by 3 µM Cact-3 produced a linear current/voltage relationship, very similar in magnitude to that produced by maximal forskolin activation. The Cact-3-induced chloride current increase was blocked by CFTR_inh_-172, a specific CFTR inhibitor [20].

### 2.3. Cact-3 Does Not Alter cAMP Level, Channel Activity of ANO1 and hERG, or Cell Viability

**Figure 3:**
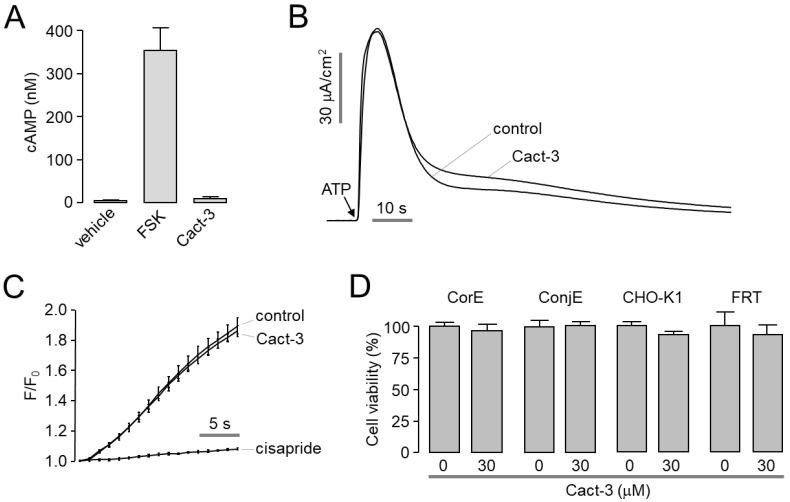
Effect of Cact-3 on cytosolic cAMP level, ANO1, hERG, and cell viability. (**A**) cAMP accumulation was measured in CHO-K1 cells. Cells were treated with forskolin (20 µM) and Cact-3 (30 µM) for 10 min (mean ± S.E., *n* = 3). (**B**) Representative traces of apical membrane currents in FRT-ANO1 cells. Cact-3 was pretreated 10 min prior to activation of ANO1 by ATP (100 μM). (**C**) Effect of Cact-3 on hERG activity was measured using FluxOR assay in recombinant HEK293 cell line expressing hERG (mean ± S.E., *n* = 6). Cact-3 (30 μM) and cisapride (50 μM, a hERG inhibitor) were pretreated for 10 min. (**D**) CorE and ConjE cells were treated with 30 µM Cact-3 for 48 h and then cell viability was assessed by MTS assay (mean ± S.E., *n* = 6).

To investigate whether Cact-3 activated the CFTR Cl^-^ channels through the elevation of the intracellular cAMP levels, we observed the effect of Cact-3 on the intracellular cAMP levels in CHO-K1 cells and discovered that it did not affect the intracellular cAMP levels (Figure 3A). To observe the effect of Cact-3 on other ion channels, including ANO1 and hERG channels, the apical membrane currents of ANO1 were measured in FTR cells expressing human ANO1; hERG K^+^ channel activity was measured using a FluxOR potassium ion channel assay in HEK293 cells expressing hERG. As shown in Figure 3B and C, Cact-3 did not alter the channel activity of ANO1 and hERG at a high concentration (30 μM). To evaluate the cytotoxic effect of Cact-3, the cell viability was measured in the corneal epithelial (CorE), conjunctival epithelial (ConjE), CHO-K1, and FRT cells. Cact-3 did not affect the cell viability of CorE, ConjE, CHO-K1, or FTR cells at 30 μM (Figure 3D).

### 2.4. Cact-3 Potently Activates Endogenous CFTR Chloride Channels and Increases Tear Volume in Mice

**Figure 4:**
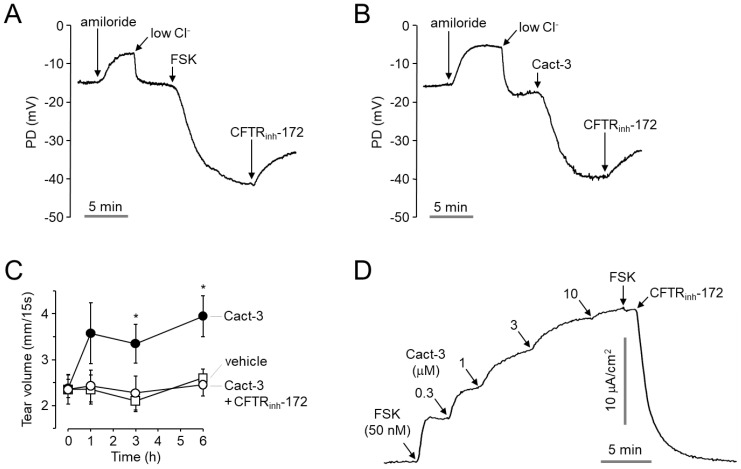
Effect of Cact-3 on mouse ocular surface PD and tear volume, and CFTR activity in primary cultured human conjunctival epithelial cells. (**A**,**B**) Representative traces of ocular surface open-circuit PD measurements. PD was measured in response to perfusion with solutions containing indicated compounds (100 μM amiloride, 20 μM forskolin, 30 μM Cact-3, and 20 μM CFTR_inh_-172). (**C**) Tear volume was measured using phenol red thread test at the indicated times after 2.5 μL of single ocular delivery of a vehicle, Cact-3 (100 µM) and CFTR_inh_-172 (20 µM), in mice (mean ± S.E., *n* = 5). (**D**) Representative trace of short circuit current in primary cultured human conjunctival epithelial cells. CFTR was weakly activated by 50 nM, enhanced by the indicated concentrations of Cact-3, and inhibited by 10 μM CFTR_inh_-172. * *p* < 0.05.

To observe the effect of Cact-3 on the CFTR channel activity on the ocular surface of a mouse, we measured the open-circuit potential difference (PD) across the ocular surface of ICR mice. As shown in Figure 4A,B, representative traces of ocular surface PD showed a small amiloride-induced depolarization followed by a low Cl^−^ perfusate-induced hyperpolarization and the subsequent activation of CFTR by forskolin (20 µM) and Cact-3 (30 µM). The application of CFTR_inh_-172 inhibited the forskolin- and Cact-3-induced hyperpolarization. To investigate the effect of Cact-3 on tear secretion in mice, the tear volume was measured using phenol red thread in mice. Cact-3 significantly increased the tear volume by >1.5 times from 3 to 6 h after the treatment and the Cact-3-induced tear volume increase was completely blocked by CFTR_inh_-172 (Figure 4C).

To investigate whether Cact-3 could activate endogenous CFTR in a human ocular epithelium, we measured the short-circuit current in primary cultured human conjunctival epithelial (HCE) cells. As shown in Figure 4D, Cact-3 significantly increased the CFTR-mediated chloride current in a dose-dependent manner and fully activated CFTR at 10 µM. The Cact-3-induced CFTR activation was completely blocked with 10 μM CFTR_inh_-172.

### 2.5. Cact-3 Improves Dry Eye Syndrome in a Dry Eye Mouse Model

**Figure 5:**
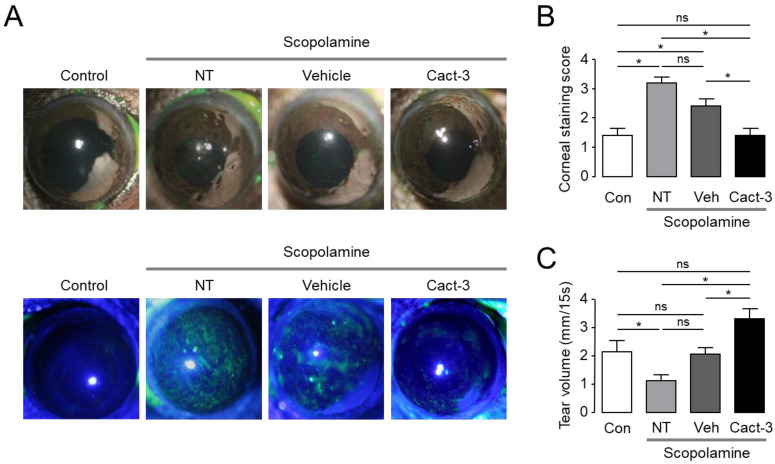
Cact-3 reduced ocular surface damage and increased tear volume in a scopolamine-induced dry eye mouse model. (**A**) Representative photographs of mouse eyes (**top**) and corneal fluorescein staining images of mice (**bottom**). Vehicle and Cact-3 were treated 3 times a day for 10 days. (**B**) Summary of corneal fluorescein staining scores (mean ± S.E., *n* = 5). (**C**) Summary of basal tear volume measured with phenol red thread test (mean ± S.E., *n* = 8). NT: not treated; ns: not significant. * *p* < 0.05.

Cact-3 was applied to the dry eye mouse model to confirm whether Cact-3 was effective in treating dry eye syndrome in the in vivo animal model (Figure 5). In a mouse model of dry eye syndrome induced by scopolamine and a dry chamber, Cact-3 was used for 10 days and it was confirmed that corneal erosion was significantly reduced compared with the vehicle group. In addition, as a result of measuring the tear volume using the phenol red thread test, it was confirmed that the amount of tear volume in the Cact-3-treated group significantly increased compared with the vehicle group.

### 2.6. Cact-3 Reduces Ocular Proinflammatory Cytokine Levels in a Dry Eye Mouse Model

**Figure 6:**
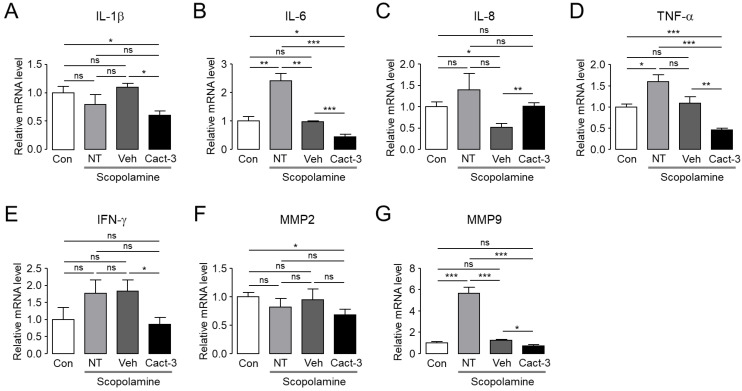
Cact-3 reduced expression of proinflammatory cytokines of cornea and conjunctiva in a scopolamine-induced dry eye mouse model. The relative mRNA expression level of IL-1β (**A**), IL-6 (**B**), IL-8 (**C**), TNF-α (**D**), IFN-γ (**E**), MMP-2 (**F**), and MMP-9 (**G**) in cornea and conjunctiva were presented. Dry eye mice were treated with vehicle or Cact-3 for 10 days whilst maintaining the dry eye condition (mean ± S.E., *n* = 4). NT: not treated; ns: not significant. * *p* < 0.05; ** *p* < 0.01; *** *p* < 0.001.

It is known that when dry eye disease occurs, an inflammation of the ocular surface is aggravated and, accordingly, the level of inflammation-related cytokines in the tear and ocular surface epithelial cells changes. Therefore, we measured the expression of various inflammation-related molecules to determine whether a treatment with Cact-3 improved the inflammation of the ocular surface in a dry eye mouse model (Figure 6). The mRNA expression of interleukin (IL)-1β, IL-6, tumor necrosis factor (TNF)-α and interferon (IFN)-γ was significantly reduced in the Cact-3-treated group compared with the vehicle group. Although the mRNA level of matrix metalloprotease (MMP)-2 was comparable, MMP-9 was significantly decreased after the Cact-3 treatment in the dry eye mouse model. Overall, we observed that a treatment of Cact-3 improved dry eye disease and decreased proinflammatory cytokines and MMPs.

## 3. Discussion

DED is one of the most complicated chronic ocular surface diseases due to the loss of tear film homeostasis and tear film hyperosmolarity, an important etiological factor in DED that causes proinflammatory signals and ocular epithelial cell damage [21,22,23]. Recent evidence has suggested that CFTR activation can rescue the hyperosmolarity of the tear film and ameliorate DED by inducing ocular surface water secretion [17,19]. CFTR is expressed in the corneal and conjunctival epithelium and induces fluid secretion from the ocular surface via chloride transport [10,24,25].

Previous studies have shown that a topical application of IBMX, a non-selective phosphodiesterase (PDE) inhibitor, increases tear secretion and decreases tear film osmolality through CFTR activation in dry eye patients, and that CF patients with defective CFTR exhibit tear film abnormalities more frequently than normal subjects [13,14,15]. Interestingly, a recent study showed that the ocular surface epithelium of human subjects had a strong CFTR channel activity, but a relatively weak CaCC and ENaC activity [16]. Therefore, CFTR activators that induce a sustained fluid secretion from the ocular surface epithelium may be more useful in the treatment of patients with dry eye than CaCC activators or ENaC inhibitors. A previous study showed that chloride transport in the corneal epithelium was synergistic with basolateral K^+^ transport [26]. Although this study did not show the effect of the CFTR activator on the basolateral K^+^ transport, the CFTR activators that can induce basolateral K^+^ transport together could induce fluid secretion more effectively.

In this study, we identified Cact-3, a novel potent activator of CFTR that exhibits a strong and sustained activation of CFTR with no cytotoxicity in human corneal and conjunctival epithelial cell lines (Figure 2 and Figure 3D). The topical application of Cact-3 on the ocular surface significantly increased the tear volume of normal mice for several hours (Figure 4C) and these results suggest that efficient tear production and the turnover of the tear film can occur via CFTR activators.

In order to be used as a drug to treat DED in patients, it is fundamentally important to show therapeutic effects in animal experiments. The current study confirmed that Cact-3 improved the signs of DED and reduced the inflammation-related markers, including cytokines and MMPs, in a dry eye mouse model that was induced by scopolamine and a controlled environment chamber. The expression of CFTR in the epithelial cells on the ocular surface has already been reported by previous studies [10]. In addition, CFTR activators such as isorhamnetin have been shown to treat DED [17,19]. Cact-3, as used in this study, has a high potential for drug development in the future because it showed a stronger treatment effect for dry eye compared with the vehicle (Figure 5 and Figure 6).

Even in a group that was treated with only the vehicle, the dry eye syndrome showed a tendency to improve compared with the not treated (NT) group. In the vehicle-treated group, the expression of IL-6 and MMP-9 was significantly decreased compared with the NT group and in the case of IL-8 and TNF-α, although not significant, there was a clear tendency to decrease. In addition, although the corneal staining score and tear volume were not statistically significant, the vehicle-treated group showed a slightly better condition compared with the NT group. The vehicle contained components of artificial tears such as polysorbate 80; thus, even the vehicle alone showed a therapeutic effect in dry eye mice. However, the therapeutic effect on dry eye was much better in the Cact-3-treated group than in the vehicle-treated group (Figure 5 and Figure 6).

When comparing the expression of proinflammatory cytokines between the control group and the NT group, IL-1β, IL-8, and IFN-γ did not show a significant difference, but the Cact-3-treated group showed a significant decrease (Figure 6). It is difficult to know exactly why there were no significant differences between the control and NT groups, but it is possible that the mice were exposed to a dry breeding environment. As shown in Figure 5, the Cact-3 treatment group showed a higher tear volume than the control group; corneal erosion was also observed in the control group. IL-6, TNF-α, and MMP-9 showed a significant increase in the NT group compared with the control group; IL-8 and IFN-γ also showed a tendency to increase in the NT group compared with the control group although it did not reach a statistical significance. Unlike other cytokines, in the case of IL-8, the Cact-3-treated group showed an increased concentration compared with the vehicle-treated group (Figure 6). Although the reason was not elucidated in this study, it is possible that Cact-3 influenced the regulatory mechanisms related to IL-8 expression. Further studies on these effects are needed in the future.

Although further experiments are needed, it is encouraging that no complications were found in the mouse model treated with Cact-3. Improvements to the chemical properties and solubility of Cact-3 will increase the possibility of the development of new DED therapeutics.

In summary, a novel potent CFTR activator, Cact-3, induced the strong and sustained activation of CFTR in vitro and in vivo. Cact-3 did not alter the channel activity of CaCC and hERG or the cell viability of human corneal and conjunctival epithelial cell lines. Notably, the topical application of Cact-3 resulted in a marked increase in tear volume and a decreased expression of proinflammatory cytokines as well as ocular surface damage in a scopolamine-induced dry eye mouse model. These results suggest that Cact-3 is a useful pharmacological tool for CFTR studies and may be a potential development candidate for the treatment of DED.

## 4. Materials and Methods

### 4.1. Cell Culture and Cell Lines

Chinese hamster ovary (CHO)-K1 cells expressing a human wild-type (WT)-CFTR with a halide sensor YFP-F46L/H148Q/I152L were grown in Dulbecco’s Modified Eagle’s Medium supplemented with 10% FBS, 2 mM glutamine, 100 units/mL penicillin, and 100 µg/mL streptomycin. Fisher rat thyroid (FRT) cells expressing human WT-CFTR with a halide sensor YFP-H148Q/I152L were generously provided by Dr. Alan Verkman (University of California, San Francisco) and grown in F12 Modified Coon’s Medium supplemented with 10% FBS, 2 mM glutamine, 100 units/mL penicillin, and 100 µg/mL streptomycin. Immortalized human corneal epithelial (CorE) cells were grown in a bronchial epithelial cell growth medium (Lonza, Basel, Switzerland) with all the supplements. Immortalized human conjunctival epithelial (ConjE) cells were grown in a corneal epithelial cell medium (ScienCell, Carlsbad, CA, USA) with all the supplements.

The primary culture of human conjunctival epithelial cells was performed as reported previously with a few modifications [27]. The conjunctival specimen was washed twice with PBS and chopped with fine ophthalmologic scissors. The fragments were incubated for 30 min at 37 °C with 0.1% Protease (Sigma-Aldrich, St Louis, MO, USA) in a 1:1 mixture of DMEM and Ham’s nutrient mixture F12 (DMEM/F12, Gibco, Grand Island, NY, USA) supplemented with 1% penicillin–streptomycin (P/S, Gibco). The sample was centrifuged for 3 min at 1000× *g* rpm and the supernatants was discarded. It was then incubated for 1 h at 37 °C with 0.2% collagenase (Sigma-Aldrich) in DMEM/F12 supplemented with 1% P/S. The suspension was filtered through a 70 μm cell strainer and centrifuged for 3 min at 1000× *g* rpm. The cells were seeded and grown in a bronchial epithelial growth medium (BEGM) BulletKit^TM^ (Lonza) supplemented with 1% P/S. After the cultures reached a 70~80% confluence, the cells were subcultured and grown in Snapwell (Corning Inc., Corning, NY, USA) coated with collagen I (Corning Inc.) in a 1:1 mixture of DMEM and BEGM BulletKit^TM^ supplemented with 0.15 mg/mL bovine serum albumin (Sigma-Aldrich) and 10 μM retinoic acid (Sigma-Aldrich).

### 4.2. Materials and Reagents

Forskolin and other chemicals, unless otherwise indicated, were purchased from Sigma-Aldrich (St. Louis, MO, USA). Cact-3 was purchased from ChemDiv (San Diego, CA, USA). The compound collections used for screening included 50,000 synthetic small molecules from ChemDiv. The compounds were maintained as dimethylsulfoxide stock solutions.

### 4.3. YFP Fluorescence Quenching Assay

CHO-K1 cells expressing wild-type CFTR with the halide sensor YFP-H148Q/I152L were plated in 96-well black-walled microplates (Corning Inc., Corning, NY, USA) at a density of 2 × 10^4^ cells per well. The CHO-WT-CFTR-YFP cells were incubated for 48 h at 37 °C. The assays were performed using a FLUOstar Omega microplate reader (BMG Labtech, Ortenberg, Germany) and MARS Data Analysis Software (BMG Labtech). Each well of a 96-well plate was washed 3 times in PBS (200 µL/wash). A total of 100 µL PBS was added to each well. The test compounds (1 µL) were added to each well. After 10 min, the 96-well plates were transferred to the microplate reader and preheated to 37 °C for the fluorescence assay. Each well was individually assayed for CFTR-mediated I^-^ influx by continuously recording the fluorescence (200 ms per point) for 2 s (baseline); 100 µL of a 140 mM I^−^ solution was then added at 2 s and the YFP fluorescence was recorded for 14 s. The initial iodide influx rate was determined from the initial slope of the fluorescence decrease by a non-linear regression following the infusion of iodide.

### 4.4. Transepithelial Electrical Measurements

Snapwell inserts containing CFTR-expressing FRT and a primary culture of human conjunctival epithelial cells were mounted in Ussing chambers (Physiologic Instruments, San Diego, CA, USA). For the FRT cells, to generate the transepithelial Cl^-^ gradient, a basolateral bath was filled with a HCO_3_^−^-buffered solution containing (in mM): 120 NaCl, 5 KCl, 1 MgCl_2_, 1 CaCl_2_, 10 d-glucose, 5 HEPES, and 25 NaHCO_3_ (pH 7.4). The apical bath was filled with the half-Cl^−^ solution; 65 mM NaCl in the HCO_3_^−^-buffered solution was replaced by Na gluconate and the basolateral membrane was permeabilized with 250 µg/mL amphotericin B. For the primary cultures of the human conjunctival epithelial cells, symmetrical HCO_3_^-^-buffered solutions were used and ENaC was inhibited by a pretreatment with amiloride (100 µM). All cells were bathed for a 20 min stabilization period and aerated with 95% O_2_/5% CO_2_ at 37 °C. The apical membrane current and short-circuit current were measured with an EVC4000 Multi-Channel V/I Clamp (World Precision Instruments, Sarasota, FL, USA) and recorded using PowerLab 4/35 (AD Instruments, Castle Hill, Australia). The data were collected and analyzed with AD Instruments acquisition software Labchart Pro 7. The sampling rate was 4 Hz.

### 4.5. hERG Channel Activity

HEK293 cells stably expressing human Kv11.1 (hERG) were plated in 96-well plates and incubated for 48 h. To enhance the membrane expression of hERG, the cells were incubated at 28 °C for 4 h before a FluxOR assay. The culture medium was replaced with 80 µL/well of a FluxOR loading buffer (Invitrogen, Carlsbad, CA, USA) and the cells were incubated for 1 h at 37 °C in the dark. After the removal of the loading buffer, 100 µL of the assay buffer was added to each well. The cells were pretreated with test compounds for 10 min and then transferred to a FLUOstar Omega microplate reader (BMG Labtech). FluxOR fluorescence (excitation/emission: 490/525 nm, respectively) was recorded for 4 s before the addition of 20 µL of a stimulus buffer containing thallium ions; the fluorescence was then monitored. FluxOR fluorescence was analyzed using MARS Data Analysis Software (BMG Labtech). All buffers were prepared according to the manufacturer’s instructions.

### 4.6. Patch-Clamp

Whole-cell patch-clamp recordings were performed on CFTR-expressing CHO-K1 cells. The bath solution contained (in mM): 140 NMDG-Cl, 1 CaCl_2_, 1 MgCl_2_, 10 glucose, and 10 HEPES (pH 7.4). The pipette solution contained (in mM): 130 CsCl, 0.5 EGTA, 1 MgCl_2_, 1 Tris-ATP, and 10 HEPES (pH 7.2). The pipettes were pulled from borosilicate glass and had resistances of 3–5 MΩ after fire polishing. The seal resistances were between 3 and 10 GΩ. After establishing the whole-cell configuration, CFTR was activated by forskolin and/or Cact-3. Whole-cell currents were elicited by applying hyperpolarizing and depolarizing voltage pulses from a holding potential of 0 mV to potentials between −80 and +80 mV in steps of 20 mV. The recordings were made at room temperature using an Axopatch-200B (Axon Instruments). The currents were digitized with a Digidata 1440A converter (Axon Instruments), filtered at 5 kHz, and sampled at 1 kHz.

### 4.7. cAMP Measurement

CHO-K1 cells grown on 12-well culture plates were washed 3 times with PBS and then incubated in PBS containing 100 µM 3-isobutyl-1-methylxanthine (IBMX) at 37 °C for 5 min. The cells were treated with the test compounds and incubated for 10 min at 37 °C. After a 10 min incubation, the cells were washed with cold PBS and cytosolic cAMP was measured using a cAMP immunoassay kit (Parameter cAMP Immunoassay Kit; R&D Systems, Minneapolis, MN) according to the manufacturer’s protocol.

### 4.8. Ocular Potential Difference (PD) Measurement

Male ICR mice weighing 30 g were studied at the age of 8 weeks. The animal study protocols were approved by the Institutional Animal Ethics Committee of Yonsei University. The mice were anesthetized with 2,2,2-tribromoethanol (avertin, 125 mg/kg intraperitoneal; Sigma-Aldrich, St. Louis, MO, USA), with additional avertin injected during the experiments to maintain the anesthesia. The mice were immobilized during the experiment with a custom-built lift and the face was positioned so that the eye was facing upward. The eyes were kept hydrated with a regular solution containing (in mM): 150 NaCl, 5 KCl, 1 MgCl_2_, 1 CaCl_2_, 10 d-glucose, and 10 HEPES (pH 7.4). The tip of the perfusion tube was positioned ~1 mm above the ocular surface of the mice. The body temperature of the mice was kept at 37 ± 1 °C by putting a heating pad underneath the lift. For measuring the potential difference at the ocular surface, a 1 M KCl agar bridge connected to an Ag/AgCl electrode and a high-impedance digital voltmeter (6 1/2 Digit Multimeter; Picotest, Phoenix, AZ, USA) was positioned alongside with the tip of the perfusion tube. The reference electrode, which consisted of a second Ag/AgCl electrode with a 1 M KCl agar bridge and a 320 mOsm saline-filled syringe needle, was inserted into the subcutaneous tissue at the back. After the initial measurement of the baseline, 100 µM amiloride was applied and the regular solution was replaced with a low Cl^-^ solution containing (in mM): 155 Na^+^ gluconate, 5 K^+^ gluconate, 1 MgCl_2_, 1 CaCl_2_, 10 d-glucose, and 10 HEPES (pH 7.4). To observe the chloride channel activities on the mouse ocular surface, 20 µM forskolin, 30 µM Cact-3, and 20 µM CFTR_inh_-172 were added according to the solution in the reservoir. The potential difference at the mouse ocular surface was recorded using M35XX software (Picotest, Phoenix, AZ, USA) at a rate of 250 µS per point.

### 4.9. Tear Volume Measurement

Female C57BL/6 mice were studied at the age of 7 weeks. The animal study protocols were approved by the Ildong Pharmaceutical Co., Ltd. (Hwaseong, Korea). Institutional Animal Care and Use Committee (IACUC). The tear volume was measured using phenol red threads (Zone-Quick, Oasis Medical, San Dimas, CA, USA) by placing them in the lateral canthi of the mice for 15 s using forceps. The results were obtained by measuring the length of the phenol red thread turning red by tears. Serial measurements of the tear volume were performed after the topical application of 2.5 μL eye drops containing 0.3% carboxymethylcellulose, 0.015% benzalkonium chloride, 1% DMSO, and 100 μM Cact-3 in saline with or without 20 μM CFTR_inh_-172 for 6 h.

### 4.10. Dry Eye Mouse Model

This study was approved by the Committee on Animal Research at Yonsei Medical Center. All animal studies were performed in accordance with the Yonsei Medical Center Animal Research Guidelines, which adhere to the standards articulated in the Association for Assessment and Accreditation of Laboratory Animal Care International (AAALAC) guidelines and the ARVO Statement for the Use of Animals in Ophthalmic and Vision Research. Dry eye mouse models were developed as described previously [19]. Eight-week-old C57BL/6 female mice, purchased from Orient Bio Inc. (Sungnam, Korea), were used for this experiment. Experimental dry eye was induced by the subcutaneous injection of 5 mg/mL scopolamine hydrobromide (Sigma-Aldrich, St. Louis, MO) three times a day in a standard desiccating environment created by placing the mice in a chamber with a continuous air flow (15 L/min) in a room at 25 °C with an ambient humidity of 35%. Fourteen days after the initiation of the experimental dry eye, the mice were treated with or without 5 μL eye drops that contained Cact-3 (48 μM) in 1% polysorbate 80 in phosphate 4 times a day for 10 days. Ten days after the treatment, the measurement of tear volume using the phenol red thread test (Zone-Quick), corneal staining score, and relative mRNA level were performed.

### 4.11. Corneal Fluorescein Staining

After approximately 10 µL of fluorescein solution was applied to the lateral conjunctival sac of the mice, they were rinsed with saline. The eyes were examined for corneal staining under a cobalt blue light. The corneal staining score was measured using the standardized Oxford grading system.

### 4.12. Quantitative PCR Analysis

The mRNA expressions of IL-1β, IL-6, IL-8, TNF-α, IFN-γ, MMP-2, and MMP-9 in the cornea of the dry eye model-implemented mice were measured by qPCR as described previously [19]. RNA was isolated using a Tri-RNA reagent (FAVORGEN, Ping-Tung, Taiwan) and 1 μg of RNA was used to synthesize the complementary DNA (cDNA) using an RNA to cDNA EcoDryTM premix (TaKaRa, Shiga, Japan) according to the manufacturer’s protocol. The relative mRNA levels were measured in ViiA7 (Applied Biosystems, Foster City, CA, USA) using a SYBR Green PCR Master Mix (Applied Biosystems). The sequences of the primers used in this study are shown in Table 1.

### 4.13. Statistical Analysis

The results of the multiple experiments were presented as the means ± S.E. The statistical analysis was performed with a Student’s *t*-test or by an analysis of variance as appropriate. A value of *p* < 0.05 was considered to be statistically significant.

## Figures and Tables

**Table 1:** Primers used for the quantitative PCR analysis.

Gene	Forward (5′-3′)	Reverse (5′-3′)	Product Size (bp)
GAPDH	AACGACCCCTTCATTGACCT	ATGTTAGTGGGGTCTCGCTC	155
IL-1β	ACTCATTGTGGCTGTGGAGA	TTGTTCATCTCGGAGCCTGT	199
IL-6	CTGCAAGAGACTTCCATCCAG	AGTGGTATAGACAGGTCTGTTGG	131
IL-8	CCCTGTGACACTCAAGAGCT	CAGTAGCCTTCACCCATGGA	190
TNF-α	AGCACAGAAAGCATGATCCG	CGATCACCCCGAAGTTCAGT	166
IFN-γ	TTCTTCAGCAACAGCAAGGC	ACTCCTTTTCCGCTTCCTGA	156
MMP-2	CGATGTCGCCCCTAAAACAG	GCATGGTCTCGATGGTGTTC	176
MMP-9	AAAACCTCCAACCTCACGGA	GTGGTGTTCGAATGGCCTTT	190

## Abbreviations

CFTR	Cystic fibrosis transmembrane conductance regulator
Cact-3	7-(3,4-dimethoxycyclohexyl)-N-(4-ethoxyphenyl)pyrazolo [1,5-α]pyrimidine-2-carboxamide
ANO1	Anoctamin 1
DED	Dry eye disease
ENaC	Epithelial Na^+^ channel
hERG	Human ether-a-go-go-related gene
PD	Potential difference
HCE	Human conjunctival epithelial
FSK	Forskolin
